# Recognizing short coding sequences of prokaryotic genome using a novel iteratively adaptive sparse partial least squares algorithm

**DOI:** 10.1186/1745-6150-8-23

**Published:** 2013-09-25

**Authors:** Sun Chen, Chun-ying Zhang, Kai Song

**Affiliations:** 1 School of Chemical Engineering and Technology, Tianjin University, Tianjin 300072, China

**Keywords:** Iteratively adaptive SPLS, Short coding sequence, Prokaryotic genome

## Abstract

**Background:**

Significant efforts have been made to address the problem of identifying short genes in prokaryotic genomes. However, most known methods are not effective in detecting short genes. Because of the limited information contained in short DNA sequences, it is very difficult to accurately distinguish between protein coding and non-coding sequences in prokaryotic genomes. We have developed a new Iteratively Adaptive Sparse Partial Least Squares (IASPLS) algorithm as the classifier to improve the accuracy of the identification process.

**Results:**

For testing, we chose the short coding and non-coding sequences from seven prokaryotic organisms. We used seven feature sets (including GC content, Z-curve, etc.) of short genes.

In comparison with GeneMarkS, Metagene, Orphelia, and Heuristic Approachs methods, our model achieved the best prediction performance in identification of short prokaryotic genes. Even when we focused on the very short length group ([60–100 nt)), our model provided sensitivity as high as 83.44% and specificity as high as 92.8%. These values are two or three times higher than three of the other methods while Metagene fails to recognize genes in this length range.

The experiments also proved that the IASPLS can improve the identification accuracy in comparison with other widely used classifiers, i.e. Logistic, Random Forest (RF) and *K* nearest neighbors (KNN). The accuracy in using IASPLS was improved 5.90% or more in comparison with the other methods. In addition to the improvements in accuracy, IASPLS required ten times less computer time than using KNN or RF.

**Conclusions:**

It is conclusive that our method is preferable for application as an automated method of short gene classification. Its linearity and easily optimized parameters make it practicable for predicting short genes of newly-sequenced or under-studied species.

**Reviewers:**

This article was reviewed by Alexey Kondrashov, Rajeev Azad (nominated by Dr J.Peter Gogarten) and Yuriy Fofanov (nominated by Dr Janet Siefert).

## Background

Small proteins have recently been discovered to play important roles in biological functions. Such discovery implies that the short protein coding sequences are worthy of much more research [1-3]. Significant efforts have been made previously to address the problem of short gene identification. Gao and Zhang [4] evaluated 19 feature extraction algorithms. They preferred the Z-curve methods for analyzing the short human sequences while considering both the recognition accuracy and the computational simplicity. In addition to Gao and Zhang’s work, Song et al. assessed eight state-of-the-art linear and kernel-based supervised pattern recognition techniques [5]. Saeys et al. [6] developed and compared complementary sequence features with several models in coding protein prediction (CPP) problems of animals, plants, Fungi, and Apicomplexa. Various classifiers and new feature extraction methods have also been exploited or developed for short gene identification [7-11].

In addition to these works on eukaryotic genomes, methods were also developed for short prokaryotic gene identification. The GeneHacker program was proven quite reliable in identifying short genes (those shorter than 300 nt) in cyanobacterial genomes [12]. The Metagene program was developed in 2006 by Noguchi et al. It was used to derive the model for 116 bacterial and 15 archaeal species to distinguish coding and non-coding DNA sequences [8]. The GeneMarkS program was shown to outperform other existing methods in identifying short genes in the *E. coli* genome [13]. Orphelia is a program for predicting protein coding genes in short fragments with unknown phylogenetic origin [14], as is the EasyGene [15] program.

**Figure 1:**
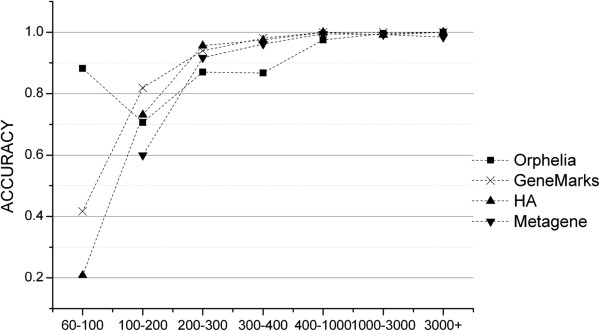
Accuracies of Four Programs to detect short genes.

However, due to the limited information provided by the very short DNA sequences, most current methods are limited in their abilities to detect very short genes. We used the short protein coding sequences of *E. coli* K-12 MG1655 to evaluate the ability of GeneMarkS [16], Metagene [8], Orphelia [14] and Heuristic Approach (HA) [17] programs in finding short genes. It can been seen in Figure 1 that gene prediction performance decreases with length and drops sharply for fragments shorter than 200 nt. The results clearly illustrated that prediction of short genes are beyond the detection ability of these tools.

Some research studies have also been made by Goli and Nair [3]. They combined various properties as the features of short DNA sequences. They then employed a Fast Correlation Based Feature Selection (FCBFS) technique [18] for dimension reduction. They also used the AdaBoost.M1 method in conjunction with the Random Forest (RF) method as the classifier. This resulted in a sensitivity of 94.77% and a specificity of 90.06% on short gene classification. Although the accuracy indicated above was somewhat improved, it is still not high enough [11,19].

Because of the limited length of short DNA sequences, it is difficult to extract features containing enough useful information. For certain feature extraction methods, the shorter the length of DNA sequences, the larger the number of the uninformative features. For the famous *Z*-curve method [20,21] for example, the shorter the length of DNA sequences, the lower the frequencies of certain tri-nucleotide patterns. Therefore, as more elements approach zero, the more they become uninformative features. The same is true of the *k*-mer frequencies approach and the Rho statistic approach. The features of both of them are calculated from nucleotide frequencies. Consequently, the important features are deemphasized by the unimportant data. The methodology of some feature extraction methods (Z-curve, etc.) indicates there are strong multi-collinear relationships among the features [3]. The classifiers for short sequence recognition problems must be influencial in handling the above mentioned adverse factors inherent in the original data set. Hence, we proposed a novel Iteratively Adaptive Sparse Partial Least Squares (IASPLS) method aimed at improving the prediction accuracy without increasing computational or memory cost. IASPLS can iteratively adapt the penalty parameters of the Singular Value Decomposition (SVD) step according to the importance of the variables. From the results of the experiments, we found that IASPLS achieved better prediction accuracy than the regular SPLS.

We chose the short coding and non-coding sequences from seven prokaryotic organisms as the samples to test the performance of the proposed method. Those are *E. coli* K-12 MG1655, *E. coli* UT189 (UPEC), *Buchnera aphidicola* 5A, *Enterobacter* 638, *K. pneumoniae* 342 and *Y. pestis* KIM 10, *Bacillus subtilis* 168.

Compared with GeneMarkS, Metagene, Orphelia, and Heuristic Approach methods, our model achieved better prediction performance in short gene classification. On the very short genes (<100 nt), it provided sensitivity as high as 83.44% and specificity as high as 92.8%. These values are two or three times higher than three of the other methods while Metagene even fails to recognize genes in this length range.

When combined with a voting system, the IASPLS classifier improved the classification specificity to 94.13% compared with Goli and Nair’s method.

In comparison with the Random Forest (RF) and the *K*-Nearest Neighbors (KNN) algorithms, IASPLS improved the prediction accuracy by 5.90% and 7.23%,respectively.

Since IASPLS is a linear method, it can reduce the calculation time by as much as ten times compared to RF or KNN. The application of IASPLS strongly confirmed the theoretical findings. Another advantage of this method is that neither the well-characterized biological features nor the parameter optimization methods are needed. Thus use of IASPLS is very effective and practical for predicting short genes of newly-sequenced or under-studied species. In this study we (1) presents the improved prediction results of IASPLS to classify short prokaryotic genes (2) describes the IASPLS algorithm in details.

## Results and discussions

The details of the IASPLS algorithm to classify short prokaryotic genes are described in the methods section. In our experiments, the prediction performance of IASPLS was evaluated using seven prokaryotic organisms whose data could be obtained from the IMG database. We also showed that IASPLS provided better prediction accuracy than Goli and Nair’s method. At last, the comparison results of IASPLS with other famous classifiers showed the superiority of it obviously.

### Comparison with other prokaryotic gene finding tools

Since evaluation of the performance of the proposed method requires comparisons with other available methods, we compared the performance of our algorithm with those of other popular existing methods with different length classes, i.e., GeneMarkS, Heuristic Approach (HA), Metagene and Orphelia. GeneMarkS utilizes a heuristic approach that builds a set of Markov models using a minimal amount of sequence information [16]. HA is a developed version which uses the linear functions between nucleotide frequencies and genomic GC content to reconstruct the Markov models in order to find genes in different organisms [17]. MetaGene gives all possible ORFs (Open Reading Frames) of the input sequences, and then uses the log-odds scoring scheme to score them by their base compositions and lengths [8]. Orpehelia is based on a kind of machine learning algorithm developed from an artificial neural network. It extracts several sequence features including monocodon usage, dicodon usage and translation initiation sites etc. Orpehelia then computes a posterior probability of an ORF to encode a protein [14]. The four programs can be run through their websites. The URLs of the websites of these four algorithms are shown in the Additional file 1.

The sequences were from seven organisms mentioned in Section Databases and Features. Since we were interested in evaluating the accuracy of prediction algorithms for short genes, all sequences were divided into length classes: [60–100 nt), [100–200 nt), [200–300 nt), [300-400 nt]. Ultimately, we obtained the 4 different datasets shown in Table 1. To obtain a balanced dataset for each length class, we used the same number of negative and positive examples. Then we applied a 5-fold cross-validation method to test them.

**Table 1:** Datasets of the organisms

**Length distribution**	**Positive samples**	**Negative samples**
[60,100)	705	3403
[100,200)	1693	5657
[200,300)	2603	2728
[300,400]	2132	1372

**Table 2:** The best recognition results obtained by different methods*

**Length**	**[60,100)**		**[100,200)**		**[200,300)**		**[300,400]**	
	**S**_**n**_**(%)**	**S**_**p**_**(%)**	**S**_**n**_**(%)**	**S**_**p**_**(%)**	**S**_**n**_**(%)**	**S**_**p**_**(%)**	**S**_**n**_**(%)**	**S**_**p**_**(%)**
Orphelia	90.21	22.13	60.07	58.90	83.57	83.65	81.97	90.87
GeneMarks	31.91	59.48	85.17	63.51	95.34	64.84	98.08	61.44
HA	16.60	79.25	76.43	74.70	94.48	74.41	96.96	71.64
Metagene	#	#	54.45	57.23	88.70	55.64	95.29	70.84
IASPLS	83.44	92.80	84.57	84.92	94.91	95.32	97.82	97.50

* ‘#’ represents there is no prediction result.

**Figure 2:**
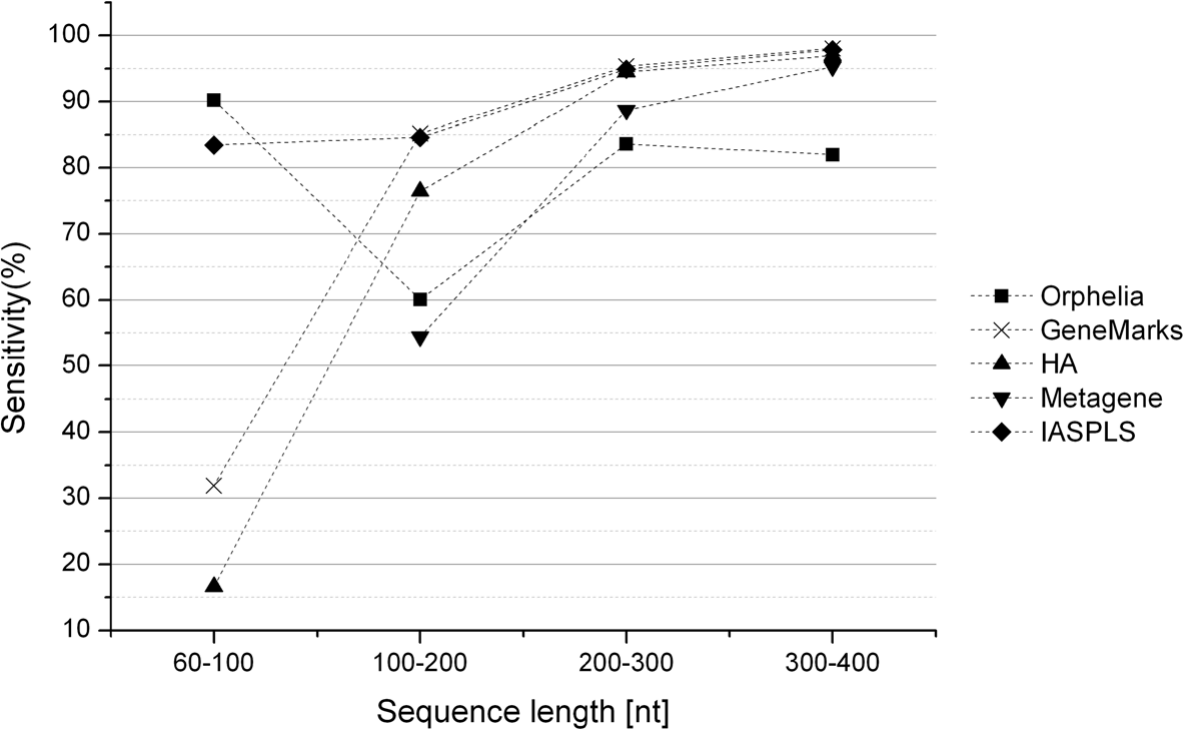
Sensitivities of the Five Prediction Programs.

**Figure 3:**
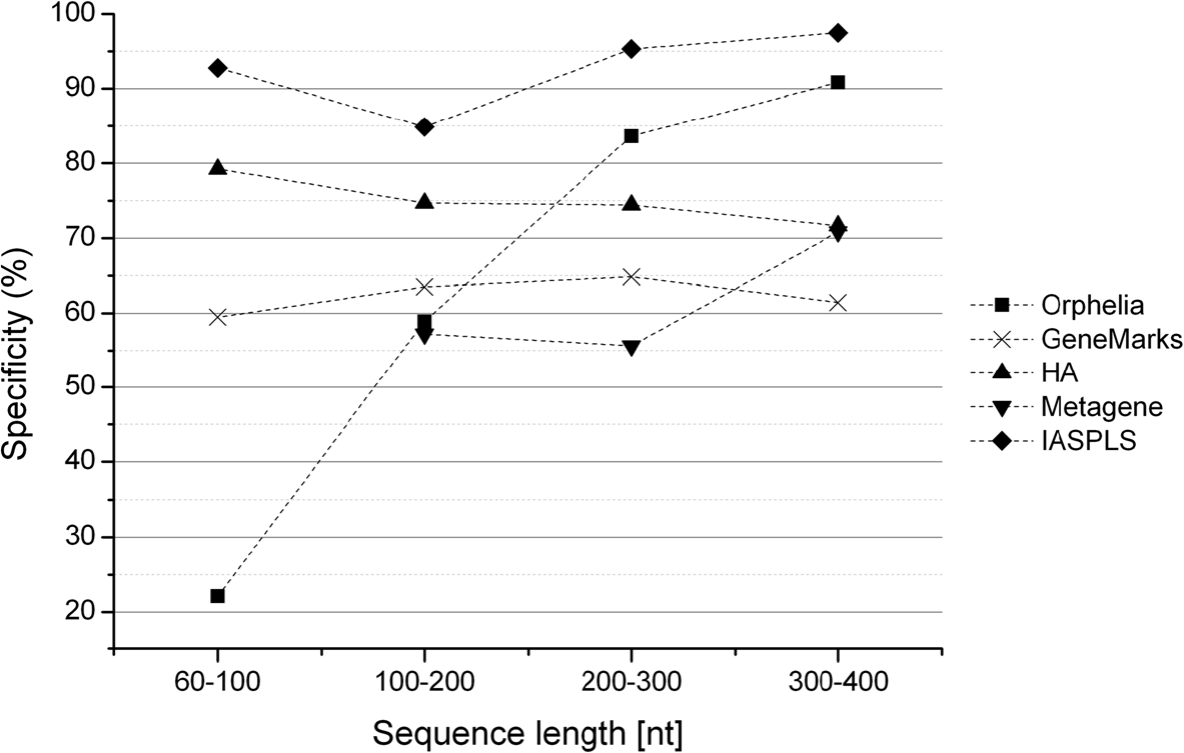
Specificities of the Five Prediction Programs.

The results of the comparison were given in Table 2. We also calculated the average values of the sensitivity and specificity of different length groups. The graphs of the sensitivity and specificity vs. sequence lengths are shown in Figures 2 and 3.

When we focused on the very short length group ([60–100 nt)), we noted that our model outperformed the other four models. Metagene was unable to identify the very short sequences demonstrating that this program is inapplicable for this length range. Orphelia achieved the highest sensitivity, 90.21%, but the lowest specificity, 22.13%. GeneMarkS and HA both yielded very poor sensitivity (31.91% and 16.60%) and specificity (59.48% and 79.25%) measurements. Our model, however, presented a better prediction performance in both sensitivity (83.44%) and specificity (92.88%). This illustrates that prediction performance of the very short coding sequences can indeed be improved markedly by IASPLS.

For the length groups ([100-200 nt), [200–300 nt) and [300–400 nt]), shown in Figure 2, sensitivities of all methods increased with sequence length. GeneMarkS had the highest sensitivities, but its specificities are not acceptable. The same phenomenon was also shown by HA. In comparison to GeneMarkS and HA, IASPLS had a higher specificity; more than 20%. Orphelia presented good specificities but unacceptable sensitivities in these length ranges. Table 2 shows that IASPLS has better performance in both sensitivity and specificity than Orphelia and Metagene.

In conclusion, GeneMarkS provided outstanding sensitivities but poor specificities for most sequence lengths longer than 100 nt. Orphelia provided an outstanding sensitivity for very short sequence (shorter than 100 nt). IASPLS consistently provided the best prediction performance in both sensitivity and specificity across all the sequence lengths.

### The performance comparison between IASPLS and Goli and Nair’s method

In 2010, Goli and Nair (GN) assembled six prokaryotic organisms for the purpose of predicting short coding sequences. They defined short coding sequences as fragments in length between 60 nt and 400 nt [3]. We used the same samples and features as GN used. The samples were the short coding and non-coding sequences of the following six organisms: *E. coli* K-12 MG1655, *E. coli* UT189 (UPEC), *Buchnera aphidicola* 5A, *Enterobacter* 638, *K. pneumoniae* 342 and *Y. pestis* KIM 10. The whole data set included 4907 coding and 3736 non-coding sequences. We used two-thirds of the coding and non-coding samples as the training set and the rest of the samples as the testing set just as GN did. We used the majority voting strategy as mentioned above. We also applied the 5-fold cross-validation method in the training step as GN did.

The best prediction results comparing the IASPLS and of GN’s method are shown in Table 3. Table 3 shows that there are improvements of 4.14% in specificity, 2.65% in sensitivity and 3.35% in ACC by using IASPLS. MCC was also improved as much as 0.06 by using IASPLS. MCC is a balanced measurement considering the different sizes of classes. The demonstrated improvement in the accuracy confirms the effectiveness of our method.

**Table 3:** Performance comparison of IASPLS with Goli and Nair’ method*

**Method**	**Results on test set**			
	**S**_**n **_**(%)**	**S**_**p **_**(%)**	**ACC (%)**	**MCC**
Goli and Nair’ method	91.26	89.89	90.67	0.81
IASPLS	**93.91**	**94.13**	**94.02**	**0.87**

*The better results between these two algorithms evaluated here were shown in boldface.

IASPLS is a linear method while GN is a combination of AdaBoost.M1 and Random Forest methods. IASPLS does not require optimization of parameters while GN requires several parameters to be optimized. It is for this reason that IASPLS has superiority in complexity. Such superiority is definitely helpful in saving computer time and memory cost.

### Comparison with other widely used classifiers

To further verify the prediction accuracy of IASPLS, we compared it with other widely used classifiers, i.e., Logistic, *K*-Nearest Neighbors (KNN) and Random Forest (RF). All of these methods have been successfully applied in solution of biological problems [3,8,22,23]. Considering the number of the samples and variables for KNN, *k* was selected from the set {1,3,5} using a three-fold cross-validation method [24]. Then for RF, the number of trees was chosen as 500 [25]. The voting process was repeated 20 times to reduce the bias caused by random partition of the training samples. The average values of the 20 voting processes were used as the final results.

The best prediction results of the four methods are listed in Table 4. The results show that IASPLS obtained the highest sensitivity (94.12%), specificity (94.15%), accuracy (94.14%) and MCC (0.88). The Logistic model showed an excellent calculation speed of 50.3 s. Its accuracy, however, was unsatisfactory. There was also a very poor tradeoff between sensitivity and specificity. Accuracy obtained in using IASPLS was improved 5.90% and 7.23% respectively in comparison with RF and KNN.

**Table 4:** The best results obtained by different classifiers*

**Classifiers**			**Results**		
	**S**_**n**_**(%)**	**S**_**p**_**(%)**	**ACC(%)**	**MCC**	Time(seconds)^**^
IASPLS	**94.12**	**94.15**	**94.14**	**0.88**	151.2 s
Logistic	93.58	87.72	91.05	0.82	**50.3 s**
SPLS	93.14	93.48	93.29	0.86	146.2 s
KNN(k = 1)	89.33	83.73	86.91	0.73	1584.1 s
Random Forest(trees = 500)	88.65	87.71	88.24	0.76	1646.5 s

*The best results among the algorithms evaluated here were shown in boldface.

**Time was the computational time of one round of training-testing process.

Calculation time per round for IASPLS was about 150 s compared with 1500 s for KNN or RF^a^. It is necessary to run the program dozens of times to optimize the parameters for each set of data. Therefore, considering both prediction performance and computer time requirement, IASPLS application is highly preferable in solving short coding sequence recognition problems.

## Methods

### The iteratively adaptive sparse partial least squares algorithm

Due to the superiority of visualization and dimension reduction, partial least squares (PLS) algorithm has been widely used in analyzing biological problems [5,26,27]. PLS can successfully handle the noise and multi-collinearity inherent in the raw data by extracting orthogonal Latent Variables (LVs) from them. PLS, being a linear algorithm, has an exceptional property for saving calculation cost over any non-linear method. More details about PLS can be found in the Additional file 1.

Sparse Partial Least Squares (SPLS) algorithm, a generalized algorithm of PLS, possesses the above mentioned superiorities of PLS. The introduced penalties in the Singular Value Decomposition (SVD) step of PLS allow the SPLS to eliminate the low Signal-to-Noise-Ratio and uninformative variables to a certain extent. The methodology of the SPLS is as follows. More details can be found in the Additional file 1.

For regular PLS, ***u*** and ***v***, the weighting vectors of ***X*** and ***Y***, respectively, can be obtained through the SVD analysis of ***M***, where ***M*** = ***X***^T^***Y***. The criterion for extracting ***u*** and ***v*** is to minimize the residual sum of squares between ***M*** and its low rank approximation:

(1) minu,vM−uv'2

where M−uv'2=∑i=1p∑j=1q(mij−uivj)2.

In order to save computational time, Anh et al. introduced regularization into the SVD step of PLS to achieve the sparseness of ***u*** and ***v***[26]. The optimization objective function therefore becomes:

(2) minu,vM−uv'2+2λ1∑i=1pui+2λ2∑j=1qvj

where *λ*_1_ and *λ*_2_ are the penalty parameters, | ∗ | is the absolute value of ∗, and *u*_*i*_ and *v*_*j*_ are the elements of ***u*** and ***v***, respectively. Through the penalties, some of elements in ***u*** and ***v*** are forced to zero because their absolute values are smaller than the soft-threshold. The immediate resulting consequence is the sparseness of ***u*** and ***v***, hence the name SPLS (Sparse PLS).

Unfortunately, the SPLS algorithm still has shortcomings. The use of constant penalty parameters to select variables in SPLS leads to the following undesirable results [28,29]:

•The contribution of the informative variables with large absolute penalty parameters, which are above the soft-threshold, may be diminished.

•Without enough prior knowledge, the useful variables may be deleted by error.

To overcome such shortcomings, we developed an Iteratively Adaptive Sparse Partial Least Squares (IASPLS) algorithm. IASPLS can adapt the values of the penalties according to the importance of the variables. Thus, we used the following objective function in the SVD step instead of using Eq. (2):

(3) minu,vM−uv'2+2λ1∑i=1pfxiui+2λ2∑j=1qvj

where *f*(***x***_*i*_) is a function of variables ***x***_*i*_, *i* = 1,2…*p*.

Theoretically, the function of the penalty parameters is two-fold: a) the weighting value of an unimportant variable should be forced to zero by use of a large enough penalty parameter. b) the contribution of an important variable should be enhanced by use of a comparatively smaller penalty parameter. Consequently, *f*(*x*_*i*_) should become a certain kind of non-increasing function to the importance of *x*_*i*_.

A classifier should be able to predict short sequences that are not limited to well-studied organisms. Hence the function/parameters which need to be optimized or selected should be as simple as possible. Although many linear and non-linear non-increasing functions can be chosen as *f*(*x*_*i*_), considering the practicality of the proposed method, we used *f*(*x*_*i*_) = 1/|*β*_*i*_|, where *β*_*i*_ is an importance index of variable *x*_*i*_. The Eq. (3) can then be written as:

(4) minu,vM−uv'2+2λ1∑i=1pωiui+2λ2∑j=1qvj

where *ω*_*i*_ = 1/|*β*_*i*_|.

Supervised pattern recognition problems may be treated as univariate regression problems. The dependent variables in these problems are defined as *y*∈{-1,+1} in two-class problems or as *y*∈{1, 2, …, *G*} in multi-class problems. Here, *G* is the number of classes. In this instance, because there is only one element in ***v***, the penalty parameter *λ*_2_ will be so insignificant in variable selection that it may be neglected. Eq. (4) can be further simplified as Eq. (5):

(5) minu,vM−uv'2+2λ1∑i=1pωiui

For a univariate PLS model, the absolute values of coefficients can be used to measure the relative importance of variables [30]. Accordingly, we can use $\omega_{i}=1/\left|{\overset{⌢}{b}}_{i}\right|$, where ${\overset{⌢}{b}}_{i}$ is the element of the regression coefficient matrix $\overset{⌢}{B}$, which was estimated by the Ridge Regression method.

According to the methodology of PLS, it may be noted that the latent variables are extracted one by one. The number of LVs should be optimized to get good trade-off between the extracted information and the deleted information (treated as noise). For the same variable, different numbers of LVs can lead to different coefficient values. To further improve the recognition performance, it is preferable to evaluate the variables’ importance iteratively. Accordingly, we named the proposed method: IASPLS (Iteratively Adaptive SPLS). The Pseudo-code for IASPLS can be found in Additional file 1.

### Databases and features

In our experiments, we used the short coding and non-coding sequences from seven prokaryotic organisms: the Gram-negative bacterium *E. coli* K-12 MG1655 (IMG: Gc00008), and its five related organisms *E. coli* UT189 (UPEC) (IMG: Gc00364), *Buchnera aphidicola* 5A (IMG:Gc00919), *Enterobacter* 638 (IMG:Gc00542), *K. pneumoniae* 342 (IMG: Gc00841) *Y.pestis* KIM 10 (IMG: Gc00095), and the Gram-positive bacterium *Bacillus subtilis* 168 (IMG: Gi03234). All these sequences can be obtained from IMG 4 database (http://img.jgi.doe.gov/cgi-bin/w/main.cgi) [31].

We used the following features as the variables in the short gene recognition problems:

•Z-curve features

•16 physicochemical and 49 conformational properties;

•84 *k*-mer frequencies (*k* = 1, 2 and 3) features;

•GC content, Codon Usage Bias (the frequency of optimal codons F_*op*_) and Codon Adaptation Index (CAI);

•Four base compositions at synonymous third codon positions);

•Amino acid properties (hydrophobicity and aromaticity);

•Rho statistic.

The Z-curve parameters are calculated for the frequencies of frame-dependent *k*-mers (1 ≤ *k* ≤ 3), using the Z-transform of DNA sequences, as exemplified in Song [32]. There are a total of 252 Z-curve features. The details of six other types of features can be found in Goli and Nair [3]. Using these features, DNA sequences could be transformed into a data set with 429 variables. Since there are several thousand samples, we preferred the majority voting strategy to lessen the dependence of the data-driven methods on the orders of the samples. In the training step, we randomly rearranged the training samples and divided them into *K* sub-blocks. We then used them to train *K* models to predict the labels of the testing samples. *K* should be an odd number for the majority voting strategy. Then there would be *K* predicted labels calculated from these *K* models for a given testing sequence. If more than half of the *K* predicted labels were ‘+1', the corresponding sequence could be assumed to be a coding sequence. If not, it would be assumed to be a non-coding sequence.

### The performance measurements of the classifiers

We used sensitivity (S_*n*_), specificity (S_*p*_), accuracy (ACC) and Matthew’s Correlation Coefficient (MCC) [33] as the measurements to evaluate the prediction performance of the classifiers. They can be defined as follows:

(6) $$S_{n}=\frac{\mathrm{TP}}{\mathrm{TP}+\mathrm{FN}}$$

(7) $$S_{p}=\frac{\mathrm{TN}}{\mathrm{TN}+\mathrm{FP}}$$

(8) $$\mathrm{ACC}=\frac{\mathrm{TP}+\mathrm{TN}}{\mathrm{TP}+\mathrm{FP}+\mathrm{TN}+\mathrm{FN}}$$

(9) $$\mathrm{MCC}=\frac{\mathrm{TP}\times \mathrm{TN}+\mathrm{FP}\times \mathrm{FN}}{\sqrt{\left(\mathrm{TP}+\mathrm{FN}\right)\times \left(\mathrm{TP}+\mathrm{FP}\right)\times \left(\mathrm{TN}+\mathrm{FN}\right)\times \left(\mathrm{TN}+\mathrm{FP}\right)}}$$

*TP*, *TN*, *FP*, and *FN* are fractions of true positive, true negative, false positive and false negative predictions, respectively.

The sensitivity, S_*n*_, is the proportion of sequences that are known as coding sequences and were correctly predicted as coding sequences. The specificity, S_*p*_, is the proportion of sequences that are known as non-coding sequences and were correctly predicted as non-coding sequences. The ACC is the proportion of the sequences that has been correctly predicted and the MCC is a balanced measurement of classification with different sizes of classes.

## Conclusions

In this paper, we developed a new IASPLS algorithm as a classifier to recognize short prokaryotic genes with high accuracy. To test it, we compared it with the most popular gene-finding softwares: GeneMarkS, Heuristic Approach (HA), Metagene and Orphelia. Our model achieved a significantly improved prediction performance in identification of short prokaryotic genes. Even in predicting the very short gene sequences, in the length of 60-100 nt, IASPLS provided sensitivity as high as 83.44% and specificity as high as 92.8%. Metagene fails to recognize genes in this length range. Orphelia achieved the highest sensitivity, 90.21%, but the lowest specificity, 22.13%. GeneMarkS and HA both yielded very poor sensitivity (31.91% and 16.60%) and specificity (59.48% and 79.25%) measurements.

We compared the IASPLS method with the method developed by Goli and Nair. The improvements of 4.14% in specificity, 2.65% in sensitivity and 3.35% in ACC were obtained by using IASPLS. Improvement over MCC was as much as 0.06 by using IASPLS.

The experiments also proved that the IASPLS can improve the identification accuracy in comparison with other widely used classifiers, i.e. Logistic, Random Forest and *K* Nearest Neighbors. The results show that IASPLS obtained the highest sensitivity (94.12%), specificity (94.15%), accuracy (94.14%) and MCC (0.88). In addition to the accuracy improvement, IASPLS required ten times less computer time than using KNN or RF.

The simplicity of IASPLS makes it more user friendly for biologists. Such simplicity also makes it more practical for performing research on new or under-studied genomes without any prior knowledge. IASPLS can be run on ordinary personal computers or laptops with run times of several minutes. Other algorithms may require more sophisticated computers or take longer computer times on personal computers or laptops.

## Endnote

^a^All algorithms were operated in MATLAB R2009a, the operation system was a 32-bit Windows XP, and the PC had 1.8G Core CPU with 2G RAM.

## Reviewers’ comments

### Reviewer’s report 1: Dr Alexey Kondrashov University of Michigan, United States of America

Reviewer comments:

Annotating genomes is outside of my narrow focus of expertise. Still, I believe that I can recognize an interesting algorithm, and the one proposed by the authors seems to be interesting. The general approach they use is not novel, but its application to short protein-coding regions is. The algorithm is described adequately - and the English, although not acceptable in its current form can be easily corrected. Whether this algorithm will consistently perform better than others remains to be seen, but it is worth publishing.

Quality of written English: Needs some language corrections before being published.

### Reviewer’s report 2: Dr Rajeev Azad (nominated by Dr J. Peter Gogarten): University of Connecticut, United States of America

Reviewer comments:

Significant efforts have been made previously to address the problem of short gene identification in prokaryotic genomes. Long genes are reliably detected by most current methods, however, many methods are limited in their abilities to detect short genes. Frequently used popular gene prediction programs such as GeneMark and EasyGene were developed to address the problem of spurious prokaryotic gene detection. Methods were also developed specifically to reliably detect short protein-coding genes, see for example, the paper by Yada and Hirosawa (Detection of short protein coding regions within the cyanobacterium genome: application of the hidden Markov model. DNA Res, vol. 3, pp.355-361,1996); this work describes the GeneHacker program that was found quite robust in identifying short genes (less than 300 nt) in cyanobacterial genomes. Yan et al. developed “Length-Shuffle” gene prediction program, based on a Z curve representation of DNA sequences, specifically to address the short gene problem (Bioinformatics, vol. 14, pp. 685-690). EasyGene was developed in 2003 by Larsen and Krogh, ranking the ORFs by their statistical significance, thereby reducing substantially the spurious short gene predictions (BMC Bioinformaticsvol.4, art. 21, 2003). GeneMarkS program was shown to outperform other existing methods in identifying short genes (< 300 nt) in E. coli genome (Zhu et al., BMC Bioinformatics, 8:97; 2007).

Strangely, and rather surprisingly, this paper has not included the developments and milestones accomplished in short gene prediction in the Background/Introduction section. None of the above frequently used programs was referenced. The authors have referenced only one paper on short gene prediction- a recently published work by Goli and Nair (2012). They say that “Concerning short prokaryotic gene recognition problems, however, only Goli and Nair [4] made some research studies”, which is not correct. It seems like that the authors have not done the literature survey on prokaryotic gene prediction and on efforts invested in addressing the short gene prediction problem.

***Author’s response*:** In the revised manuscript, we have done the literature survey on the prokaryotic gene prediction, especially on the short prokaryotic gene prediction problem. There is a literature review in the Background section. Beside Goli and Nair’ work, we reviewed other approaches including: GeneHacker, GeneMarkS, Metagene, Orphelia and so on. The experiment results illustrated that prediction of short genes (less than 200 nt) are beyond the detection ability of these tools. Thus, our motivation is to improve the prediction performance for the short genes.

One of the challenges in the field is the validation or evaluation of the prediction methods. The authors mention about 4907 coding and 3736 non-coding sequences that they used for validation of the proposed method. Beyond the size (< 400 nt), there is no further description on the fidelity of this dataset for validation.

There is no comparative study with the widely used prokarkotic gene prediction programs such as GeneMark, Glimmer, EasyGene and Prodigal. Does the proposed method outperform these popular programs in predicting short genes? The validation data could include the validated short E. coli genes from the EcoGene dataset and the validated short genes in B. subtilis (Zhu et al., BMC Bioinformatics, 8:97, 2007; Besemer et al., Nucleic Acids Res. vol. 29, pp. 2607-2618, 2001). In the absence of a comparative study, I am just left wondering why should the researchers use this method when there are already many robust methods for prediction prokaryotic genes including the short genes.

***Author’s response***: In the revised manuscript, we have compared our approach with four widely used prokarkotic gene prediction programs: GeneMarkS, Metagene, Orphelia, and Heuristic Approachs. The experimental results indicated that our method outperform these popular programs in predicting short genes. On the very short genes (<100 nt), it provided sensitivity as high as 83.44% and specificity as high as 92.8%. These values are two or three times higher than three of the other methods while Metagene even fails to recognize genes in this length range. The validation data include the Gram-negative bacterium *E. coli* genes and its five related organisms. To expand the application of our method, we add Gram-positive bacterium *B. subtilis* genes to the experiment dataset, and the prediction performance is outstanding.

The manuscript is laden with grammatical errors, unexplained formulas (eqns.1, 2, 3) and notations, and unexplained or unsubstantiated claims. The authors claim that “IASPLS has some other advantages, i.e. relying on the adaptation of such penalty values, not only the uninformative features could be removed successfully, but also the contributions of important features could be reasonably enhanced” but this has not been demonstrated. Given a gene, what could be uninformative features and what could be informative features within the gene and how the proposed method optimizes this information to reliable detect the short genes? The authors should have provided data to support this claim. Similar kinds of poorly explained or unexplained texts, without any supporting data, were found at many places throughout the manuscript.

***Author’s response***: In the revised manuscript, we gave a clear explanation for the formulas and notations, we also deleted the unexplained or unsubstantiated claims, and we invited a native English speaker to modify our paper. We believed that there were no grammatical errors in this paper.

Quality of written English: Not suitable for publication unless extensively edited

### Reviewer’s report 3: Prof Yuriy Fofanov (nominated by Dr Janet Siefert): Rice University, United States of America

Reviewer comments:

The manuscript entitled “Recognizing short coding sequences of prokaryotic genome using a novel iteratively adaptive sparse partial least squares algorithm” by Sun Chen, Chun-ying Zhang and Kai Song has major flaws. First the manuscript is poorly written and structured so that it was difficult to read. English need to be improved for readability and better comprehension.

***Author’s response***: We adjusted the structure of the article, and the revised paper has been corrected by a native English speaker, and we believed it is improved for readability and better comprehension.

Unfortunately, there is no appropriate overview of the related and previous work: This overview must be a reasonable description of where the advancement in field is at present. There are several other papers besides the paper by Goli and Niar in the gene recondition problem and these papers should be at least cited and what these papers have added to the filed.

***Author’s response*:** We have added a literature review on short prokarkotic gene prediction in the Background section, which including the papers from 1996 to 2011. The advancement in this field is Goli and Niar’ work which achieved a sensitivity of 94.77% and a specificity of 90.06% on short gene classification. But there is still room for improvement. Actually, the difficulty on short gene prediction is focus on the length under 200nt, thus we gave a research on the very short coding sequences under 200 nt.

Next, there is no clear explanation of the difference / novelty of the proposed approach. This is vital to show the readers what is new ad novel about this work. There needs to be a serious comparison of your findings with those from other approaches and show that results from your approach is better than the results of produced by other approaches. The statistical significance of the “improvement” of the proposed approach is questionable. Since only few genomes were used in the analysis significance may be improved by the analysis of more genomes. Unfortunately it was difficult to follow your formulas because not all the variables used in your formulas were described in the text. A much better description and reasoning behind the formulas must be included. Lastly, several references were presented in manner that could not be used to identify or even find them.

***Author’s response:*** In the revised manuscript, we gave a clear explanation of the difference of the proposed approach with the original SPLS algorithm. We gave the details of SPLS algorithm which is helpful for the readers to understand the improvement we proposed. We also compared our approach with four widely used prokarkotic gene prediction programs, and the experimental results proved that our method outperform the other four programs.

Quality of written English: Not suitable for publication unless extensively edited

Amended comments in response to the revision version:

Report form:

Need to fix some typos. I would also suggest to focus review on bacteria genes identification.

Quality of written English: Acceptable.

## Abbreviations

IASPLS: Iteratively adaptive sparse partial least squares.

## Competing interests

The authors declare that they have no competing interests.

## Authors’ contributions

The work presented here was done in collaboration between all authors. SC proposed the algorithm, developed the programs, designed and operated the experiments and contributed to the writing. CyZ developed the programs and designed and operated the experiments. KS supervised the project, co-designed the experiments and contributed to the writing. All authors perused and approved the manuscript.

## Supplementary Material

Additional file 1 **Partial least squares and sparse partial least squares: the details of partial least squares and sparse partial least squares algorithm.** Iteratively adaptive sparse partial least squares algorithm: the pseudo code of Iteratively adaptive sparse partial least squares algorithm. URLs of the websites of four algorithms: GeneMarkS, HA, Orphelia and Metagene. Click here for file
