# Supplementary material for: Recognizing short coding sequences of prokaryotic genome using a novel iteratively adaptive sparse partial least squares algorithm
Source: Biol Direct. 2013 Sep 25;8:23. doi: 10.1186/1745-6150-8-23 (PMC3852556; doi:10.1186/1745-6150-8-23)
Supplement: Additional file 1 — Partial least squares and sparse partial least squares: the details of partial least squares and sparse partial least squares algorithm. Iteratively adaptive sparse partial least squares algorithm: the pseudo code of Iteratively adaptive sparse partial least squares algorithm. URLs of the websites of four algorithms: GeneMarkS, HA, Orphelia and Metagene. [file 1745-6150-8-23-S1.docx]

**Recognizing short coding sequences of** **prokaryotic genome using a novel adaptive sparse partial least squares** **algorithm**

**SunChen, Chun-ying Zhang, Kai Song****[[1]](#footnote-2)***

*School of Chemical Engineering and Technology, Tianjin University, Tianjin, 300072, China*

**Partial least squares**

Partial Least Squares (PLS) is a popular multivariate statistical analysis tool, which has been widely applied in both regression and classification fields [[1](#_ENREF_1)]. The PLS regression method aims to describe linear relationship between input and output variables. This prediction process is achieved by extracting a set of orthogonal factors called latent variables (LVs). The basic point of the procedure is that the weights used to determine these linear combinations of the original variables are proportional to the maximum covariance among input and output variables. Then the noise and the multi-colinearity of the original data are removed by compressing the *p*-dimensional X-space into the *H*-dimensional LV-space (commonly, *H*<<*p*, where *p* is the number of the original variables and *H* is the number of the latent variables). Given the standardized input variables and the response variables, the latent variables are extracted from ***X*** and ***Y*** as following:

(1)

(2)

where ***T*=**[***t***_1_,…,***t****_H_*] is latent vector matrix，and ***C***=[***c***_1_,…,***c****_H_*], ***D***=[***d***_1_,…,***d****_H_*] are loading vector matrices. ***E*** and ***F*** are residual matrix of ***X*** and ***Y*,** respectively.

There are many methods for extracting the latent vectors [[1](#_ENREF_1)]. The following algorithm is an alternative PLS program which using the singular value decomposition (SVD) of the crossproduct ***M***=***X***^T^***Y***. Then ***X*** and ***Y*** are deflated separately.

**AlgorithmS1**. Pseudo-code for PLS.

1. ***X***_0_=***X***, ***Y***_0_=***Y***
2. **for** *h* = 1…*H*，where *H* is the number of LVs: **do**
3. Set
4. Using SVD decompose and extract the first pair of singular vector ***u***=***u***_1_ and ***v***=***v***_1_corresponding to the eigenvalue with the maximum absolute value.
5. where ***t*** is the latent variable vector of ***X***
6. where ***w*** is the latent variable vector of ***Y***
7. where ***c*** is the loading vector of ***X***
8. where ***d*** is the loading vector of ***Y***

11. **end for** h=*H*

**Sparse partial least squares**

A regularized SVD has been introduced by Anh et al. [[2](#_ENREF_2)] as a method which can perform PLS with sparse weighting vectors ***u*** and ***v***. It applied the best rank one approximation property of the SVD. The weighting vectors can be obtained through the SVD of ***M***, where ***M***=***X***^T^***Y***. Since the weighting vector ***u*** which maximizes the covariance matrix between input and output is the largest eigenvalue of ***MM***^T^. In other words, ***u*** should be the first left singular vector of SVD of ***M*** [[3](#_ENREF_3)]. Thus the criterion of finding the weighting vectors equals to minimizing the residual sum of squares between ***M*** and its low rank approximation:

(3)

where, ***u*** and ***v*** are weighting vectors of ***X*** and ***Y***, respectively. The best rank-one matrix approximation of ***M*** is the product of the first left and right singular vectors and [[4](#_ENREF_4)]. Thus and . In order to achieve sparseness on ***u*** and ***v***, some regularization penalties are introduced in these regressions. The optimization problem becomes:

(4)

where *λ*_1_ and *λ*_2_ are the penalty parameters, is the absolute value of *, here *u*_i_ and *v*_j_ are the elements of ***u*** and ***v***, respectively. Through the penalties, the elements of ***u*** and ***v***, whose absolute values are smaller than the soft-threshold, are forced to be zero. The immediate consequence arising there from is the sparseness on ***u*** and ***v***, hence the name SPLS (Sparse PLS).

With the constraint , considering the optimizing problem over ***u*** with a fixed ***v***, the minimization criterion (4) can be rewritten as

(5)

Observing that, thus

(6)

Hence, the optimization problem could be written as: .

For a fixed ***u*** with, the similar expression is: .

It is easy to prove that is the solution of minimize of [[4](#_ENREF_4)]. Therefore, the optional ***u**** and ***v**** can be obtained by iteratively finding the local optional solution. The detailed program of SPLS is as follows:

**Algorithm S2**. Pseudo-code for SPLS.

1. ***X***_0_=***X***, ***Y***_0_=***Y***
2. **for** *h* = 1*…H*, where *H* is the number of LVs: **do**
3. Set
4. Using SVD decompose and extract the first pair of singular vector ***u***=***u***_1_ and ***v***=***v***_1_corresponding to the eigenvalue with the maximum absolute value.
5. Until convergence of both ***u***_new_ and ***v***_new_ (in the first iteration ***u***_old_=*u*_1_ and ***v***_old_=***v***_1_) :

, re-normalize ***u***_new_

, re-normalize ***v***_new_

***u***_old_=***u***_new_, ***v***_old_=***v***_new_

where *λ* is the penalty parameter.

1. where ***t*** is the latent variable vector of ***X***
2. where ***w*** is the latent variable vector of ***Y***
3. where ***c*** is the loading vector of ***X***
4. where ***d*** is the loading vector of ***Y***

7. **end for**

The Pseudo-code for IASPLS

1. ***X***_0_=***X***,***Y***_0_=***Y***
2. **for** *h* = 1*…H*, where *H* is the number of LVs: **do**
3. is the coefficient matrix
5. Let
6. Using SVD to decompose and extracting the first pair of singular vector ***u***=***u***_1_ and ***v***=***v***_1_，which is corresponding to the eigenvalue with the maximum absolute value.
7. Until convergence of both ***u***_new_ and ***v***_new_ (in the first iteration ***u***_old_=*u*_1_ and ***v***_old_=***v***_1_) :

, re-normalize ***u***_new_

, re-normalize ***v***_new_

***u***_old_=***u***_new_, ***v***_old_=***v***_new_

where .is the element of and *λ* is the penalty parameter.

1. where ***t*** is the latent variable vector of ***X***
2. where ***w*** is the latent variable vector of ***Y***
3. where ***c*** is the loading vector of ***X***
4. where ***d*** is the loading vector of ***Y***

7. **end for** *h*=*H* (*H* is usually determined by cross-validation, although elsewhere an F-test is suggested [[5](#_ENREF_5), [6](#_ENREF_6)]

The re-normalization of the weighting vectors ***u*** and ***v*** in step 7 of Table 1 is very important. The comparatively uninformative elements of the weighting vectors are successfully forced to zero. The following re-normalization of the variables in step 7 leads to the re-evaluation of their importance. As a result, the contributions of important variables having the larger absolute weighting values can be enhanced.

**URLs of the websites of these four algorithms**

GeneMarkS: http://exon.gatech.edu/GeneMark/genemarks.cgi

HA: <http://exon.gatech.edu/metagenome/Prediction/>

Orphelia: <http://orphelia.gobics.de/submission>

Metagene: http://weizhong-lab.ucsd.edu/metagenomic-analysis/server/metagene/

**References**

1. Rosipal R, Krämer N: **Overview and recent advances in partial least squares**. *Subspace, Latent Structure and Feature Selection* 2006:34-51.

2. k.Anh, Lê Cao, D.Rossouw, C.Robert-Granié，, P.Besse: **Sparse PLS: variable selection when integrating omic data**. *Technical report, INRA* 2008.

3. McWilliams B, Montana G: **Sparse partial least squares regression for on‐line variable selection with multivariate data streams**. *Statistical Analysis and Data Mining* 2010, **3**(3):170-193.

4. Shen H, Huang JZ: **Sparse principal component analysis via regularized low rank matrix approximation**. *Journal of Multivariate Analysis* 2008, **99**(6):1015-1034.

5. Song K: **Recognition of prokaryotic promoters based on a novel variable-window Z-curve method**. *Nucleic Acids Res* 2012, **40**(3):963-971.

6. Song K, Zhang Z, Tong TP, Wu F: **Classifier assessment and feature selection for recognizing short coding sequences of human genes**. *J Comput Biol* 2012, **19**(3):251-260.

1. [↑](#footnote-ref-2)
